# Supervision for Certification in the Field of Applied Behaviour Analysis: Characteristics and Relationship with Job Satisfaction, Burnout, Work Demands, and Support

**DOI:** 10.3390/ijerph16122098

**Published:** 2019-06-13

**Authors:** Katerina Dounavi, Brian Fennell, Erin Early

**Affiliations:** 1 School of Social Sciences, Education & Social Work, Queen’s University of Belfast, 69-71 University Street, Belfast BT7 1HL, Northern Ireland, UK; eearly03@qub.ac.uk; 2 Administration, Ambitious College, London N15 4FY, UK; bfennell@icloud.com

**Keywords:** supervision, certification, job satisfaction, burnout, applied behaviour analysis

## Abstract

*Background*: Supervision of behavior analysts seeking certification and supervision of service delivery are key processes in the provision of quality behaviour analytic services to individuals with developmental disabilities. Our study is the first to examine international supervisory practices within the field of applied behaviour analysis. *Method*: An online survey was distributed to 92 professionals internationally, assessing supervisory practice, supervisor support, work demands, job satisfaction, and burnout. *Results*: Findings indicate high satisfaction with the supervisor and supervisory experience. Excessive work demands positively correlate with high burnout and low job satisfaction. Half of all professionals only worked with one or two clients before certification. Supervisor and collegial support seem to decrease the likelihood of suffering burnout and increase job satisfaction, although relationships were not statistically significant. *Conclusions*: Supervisor and collegial support warrant further research as protective factors. Implications for an evidence-based supervisory practice that produces ethical and competent supervisees are discussed.

## 1. Introduction

Care professions have been described as those jobs in which the professional, as the giver, and the client, as the receiver, have an asymmetrical social relationship [1,2]. This imbalanced dynamic can elevate the risk of burnout across professions such as physicians, nurses, social workers, care workers, and teachers. Burnout in the care professions can have direct consequences on the quality of care and client outcomes, and as such, research on the topic is relevant to professional practice in the various fields. As a care profession, behaviour analysis requires consideration for effects of burnout on professionals and clients equally.

Plantiveau et al. [3] found high rates of burnout among Board Certified Behavior Analysts^®^ (BCBA^®^) and Board Certified assistant Behavior Analysts^®^ (BCaBA^®^), with reports of 26% stating high emotional exhaustion, 29% reporting high levels of depersonalisation, and 50% citing a lack of accomplishment. Medical doctors have a similarly high rate of burnout, with figures of 59% and 45% for US and internationally-educated doctors, respectively [4]. Although the latter study did not address the availability of support systems, the researchers found a lower rate of burnout with an increase in the number of years of training undertaken by individuals. The former research indicates increased social support produces lower rates of burnout and increased job satisfaction. A similar rate of burnout was found among surgeons, for which the authors suggest ‘wellness promotion’, including mentoring, and a ‘non-punitive system of supports’ as mechanisms for offsetting the high incidence of burnout across the profession [5]. Typical supports currently available to medical professionals are underutilised because of fears that support users will be identified as unfit for duty.

Perceived supervisor support has been shown to predict lower levels of burnout, including decreased emotional exhaustion and depersonalisation, in a study of therapists working in Applied Behaviour Analysis (ABA) school settings [6]. Similar research in the field of nursing has confirmed the positive effects of support systems on mitigating burnout. Garrosa et al. [7] found burnout was negatively correlated with social supports for emotional exhaustion in a sample of nurses in Portugal. An unpublished doctoral dissertation [8] furthered the evidence for a significant relationship between perceived supervisor support and reduced burnout with ABA therapists working with children with autism spectrum disorders.

Garrosa and colleagues [7] found individual traits (e.g., hardy personality, locus of control, and viewing novel experiences as challenges) were predictors of the likelihood to seek social support. In other words, nurses with resiliency would seek support from others when feeling stressed by professional circumstances. Griffith et al. [9] found individuals with “wishful thinking” coping strategies (i.e., passive, emotional-based coping, external locus of control) were subject to increased levels of emotional exhaustion, depersonalisation, and general stress. However, this research, contrary to others [3,6], did not indicate a positive effect on burnout from supervisor support.

Further exploration of the role of personality traits in the mitigation of burnout and its related factors has been recently reported in the field of applied behaviour analysis. Hurt et al. [10] gathered data on core personality traits (i.e., extroversion, agreeableness, conscientiousness, neuroticism, and openness to experience) and their influence on burnout factors. The results support a partial mediation of perceived personal and professional support to overall job satisfaction in the ABA therapists surveyed. Deling [11] found emotional exhaustion, depersonalisation, and personal accomplishment were negatively affected by neuroticism and positively affected by extraversion among participant ABA tutors. Burnout is more likely in individuals with higher levels of neuroticism as a personality trait, according to this research. The predisposition to burnout found in personality traits may yet be mitigated by improved social supports for all individuals in public service fields [10,11].

The effects of support systems found among ABA therapists [3,6,8] was corroborated by research among nursing professionals [12] in a study based in the US. These findings were developed further in a study of nurses in Greece which found ‘perceived’ social supports to be more effective than ‘received’ social supports [13]. Similarly, research among care workers has also found social supports particularly effective when stress levels are high, while a ‘locus of control’ is sufficient to mediate low levels of stress [14].

### Supervision in ABA

Showing competence in the application of specific procedures does not guarantee competence in teaching these to others. For example, McGimsey et al. [15] found that students who learned how to implement time-out could not teach this to parents until they were explicitly taught consultation skills. Parsons and Reid [16] also found that only after being explicitly trained on feedback provision did supervisors improve their feedback quality. These results clearly indicate that new BCBAs^®^ who might be competent in the application of behaviour-analytic procedures would most likely not be effective at instructing others unless they have received training on supervision, very recently made mandatory by the Behavior Analysts Certification Board (BACB^®^) (BACB, [17]). This training should focus on how to use behavioural skills training with others, e.g., how to use feedback [18] during in vivo and remote supervision sessions [19], or how to adopt a structured approach to supervision [20].

Evidence-based effective methods of training professionals in the use of behaviour-analytic procedures have been well established, and should form the basis of a structured supervision curriculum that is acceptable to staff members and includes performance- and competence-based training delivered in didactic and real-life work settings [20,21,22]. This endeavour could be pursued by a variety of means, such as through the establishment of reading groups aiming to enhance knowledge among human service practitioners or through the use of technology as a cost effective means of increasing collegial support [23,24]. As supervision by certified professionals (i.e., BCBA^®^ or Board Certified Behavior Analyst Doctoral; BCBA-D^™^), as opposed to non-certified individuals, and the years of supervisor experience have both been shown to produce significantly more positive outcomes for children with autism, while small variations in typical supervision intensity of 20% of treatment hours do not seem to produce significant differences in the amount of mastered skills, supervisors should possess sufficient formal training and experience before training others [25].

Supervision practice should be primarily defined according to the function it serves. Secondarily, supervisors should decide on the form that will best suit this function [26]. For example, the aim of preparing well-skilled behaviour analysts who are competent in the contents covered in the BACB^®^ Task List [27] is best served by supervision that fulfils the BACB^®^ Experience Standards [28]. The aim of establishing peer networks that will serve as a source of social support and offer opportunities for developing valuable professional skills (e.g., public speaking, collaboration with colleagues) is best served by group supervision [29,30]. Teaching students how to best provide and receive corrective feedback in group settings can be facilitated through the use of evidence-based training models and instructive instruments, such as the Corrective Feedback Instrument-Revised [31,32]. Perceived supervisor support has been shown to correlate with perceived organisational support and lead to increased job retention [33]. As primary functions of supervision practice, social support and job retention should also be at the heart of its focus.

Sellers et al. [34] offered an overview of the seven dimensions of the BACB^®^ professional and ethical compliance code [35] that relate to supervision, reflect on how the code serves as an antecedent for supervisor and supervisee behaviour, and provide a rationale for the need to reflect upon each element of the code. Hartley et al. [36] described an apprenticeship supervision model that meets the needs of trainee BCBA^®^ while proving time and cost-effective for supervisors and organisations, while other researchers have described the evaluation of the supervisor’s effectiveness and sharing of training materials as key components of supervision [37].

Research on existing supervision practice within our field is scarce [38,39]. The lack of information on existing supervision practices and supervision outcomes has led to an urgent need for more data to support the effectiveness of specific teaching strategies and guide an ethical, empowering evidence-based supervision practice that guarantees the dissemination of scientific rigour, clinical expertise, and values-driven decision-making to future behaviour analysts. Additionally, although the criteria for required academic training in behaviour analysis are well established and periodically reviewed (for a summary, see Shook and Johnston [40]), the BACB^®^ task list [27] and BACB^®^ experience standards [28] provide only a basic overview of the contents for supervised practice. It is worth noting that instructors of BACB^®^ verified course sequences need to be certified or approved by the BACB^®^ and are in most cases academics with extensive experience in research and teaching, however supervisors of practice only need to be certified and have completed 8 h of training in supervision. In practice, this means that an entry level BCBA^®^ with little clinical experience and virtually no experience in supervising others can provide supervision to trainees. Data on the profile of supervisors and how this relates to supervision outcomes are required in order to define which elements of existing supervision practices need to be refined.

## 2. Method

The main aim of the present research was to capture culturally diverse supervision practices at an international level. This was achieved through the distribution of an online survey to professionals across the globe, through e-mail lists and social media.

### 2.1. Participants

The survey was openly distributed to professionals working across the five continents in the field of ABA at all levels of certification, i.e., Registered Behavior Technicians™ (RBT^®^), BCaBA^®^, BCBA^®^, and BCBA-D^®^. For this purpose, an invitation was circulated through relevant professional organisations, e-mail lists, and social media. For analytical purposes, those respondents who clicked the survey link and engaged only with the demographic information section, providing no data on all other sections, were removed from analysis. This study therefore reports on those respondents who completed one or more sections of the survey.

A total of 92 individuals completed the survey. Key demographic results are presented in Table 1. Of all participants, 95% were females and 5% males, with age ranging from 18 to 74 years. In line with the distribution of certificants across the globe, North America was over-represented (*n* = 46, 50%), European professionals represented the second biggest group (*n* = 35, 38%), followed by five professionals from Asia (5%), four from Oceania (4%), two from South America (2%), and none from Africa (0%).

An overwhelming proportion of participants completed a university-based verified course sequence, either in the USA, Europe, or Oceania. BCBA^®^ were the largest group, with 64 participants, representing 70% of the overall sample, followed by 18 RBT^®^ (20%), 5 BCaBA^®^ (5%), and 5 BCBA-D^®^ (5%).

### 2.2. Measures

#### 2.2.1. Demographic Characteristics

The survey included five questions, focusing on participant demographics: gender, age, certification status, country where participant currently works, and the provider of the course that allowed the participant to access credential status (e.g., university where BACB^®^ verified course sequence was completed or provider of 40 h training for RBT^®^).

#### 2.2.2. Pre-Credentialing/Pre-Certification Supervision for BCaBA, BCBA, and BCBA-D

This section collected information on numerous aspects of the supervision experience that allowed participants to access their credential status, for example, the supervision allowing participants to sit for the BCBA exam. The aspects related to supervision that were examined in this section were: (a) generic information (number of attempts before passing exam, country where fieldwork was completed, exam to which supervision gave access); (b) supervisor data (credential, whether supervisor was responsible for the supervisee and/or clients, years of experience, caseload, country where supervisor was based); (c) supervised fieldwork data (modality: whether it was completed on-site and/or remotely, completion year, whether it had an individual and/or group format, frequency, number of clients included, duration, price, number of different supervisors until completion); (d) supervision evaluation (organisation, contents, supervisor skills, focus on ethics and professional conduct, ending supervision); (e) perceived supervisor support; (f) overall evaluation of supervisor; (g) overall evaluation of supervision; (h) open ended-question allowing additional comments on any aspects not previously covered.

The section focusing on perceived supervisor support included 15 questions, with items such as ‘my supervisor strongly considered my goals and values’ and ‘my supervisor took pride in my accomplishments at work’. In line with previous studies (e.g., Eisenberger et al. [33]; Rhoades et al. [41]), we adapted and included items with a factor loading between 0.70 and 0.84 from the original Perceived Organizational Support survey that indicated the supervisor’s appreciation of contribution, acknowledgement of needs, and care for the wellbeing of the supervisee (i.e., items 3, 4, 6, 7, 8, 9, 10, 17, 20, 21, 22, 23, 25, 27, 35) [42]. A five-point Likert scale was used for participants to rate their agreement with the statements [33]. Six out of 15 items selected were negatively worded to avoid an agreement response bias.

#### 2.2.3. Job Satisfaction Survey (JSS)

This section contained all 36 items of the Job Satisfaction Survey (JSS), a survey that assesses the nine facets of job satisfaction [43,44]. Participants expressed their degree of agreement with each statement using a six-point Likert scale. Examples of questions were ‘I feel I am being paid a fair amount for the work I do’, ‘I like doing the things I do at work’, ‘I have too much paperwork’, and ‘work assignments are not fully explained’. Scores in the JSS can range between 36 and 216, with scores between 36 and 108 showing dissatisfaction, 144 to 216 satisfaction, and 108 to 144 ambivalence. The reliability of the JSS has been shown to be very satisfactory for the total scale (coefficient alpha = 0.91; [43]). Validity data arising from studies that compared some JSS subscales to corresponding subscales of other job satisfaction scales have also been satisfactory, with correlations ranging from 0.61 for the coworkers subscale to 0.80 for supervision [43]. Although we do not expect the validity of the online survey to be different from the paper version, validity analyses of the JSS when distributed online should be conducted in future research.

#### 2.2.4. Excessive Work Demands

Two items were adapted from the School Organisational Health Questionnaire-Excessive Work Demands [45,46], with participants being asked to rank their agreement with these in a six-point Likert scale. These two items (i.e., ‘there is constant pressure for me to keep working’ and ‘I am always overloaded with work’) were interspersed with items from the JSS and together with item 24 (‘I have too much to do at work’) were used to compute a single variable, labelled ‘excessive work demands’.

#### 2.2.5. Maslach Burnout Inventory for Educators

The following section included the Maslach Burnout Inventory for Educators (MBI-ES) [47,48], with original items making reference to ‘students’ being adapted to read ‘students/clients’. The MBI-ES includes 22 items and evaluates the three factors that compose burnout, emotional exhaustion, depersonalisation, and lack of personal accomplishment. Participants ranked how often they experience certain feelings in their work placement using a six-point Likert scale ranging from never to every day to (e.g., ‘I feel emotionally drained from my work’, ‘I feel I treat some students/clients as if they were impersonal objects’, ‘I feel very energetic’).

A meta-analysis of reliability data reported in the literature for the MBI showed satisfactory coefficients of 0.88, 0.71, and 0.78 for Emotional Exhaustion, Depersonalisation and Personal Accomplishment respectively. Convergent and discriminant validity have been tested and are reported in the manual [49].

### 2.3. Procedure

After receiving ethical approval, an invitation to complete an online survey was circulated to certificants/registrants working in ABA-related settings. The first section of the survey included an informed consent. In the second section, participants provided key demographic data and proceeded to the completion of the pre-certification supervision section. Following this, participants proceeded to the sections measuring job satisfaction, excessive work demands, and burnout. The survey took approximately 20 min to complete, with most sections incorporating a feature that did not allow progress to the next page unless all questions had been answered.

### 2.4. Statistical Analysis of Survey Data

Data collected from demographic information, the pre-certification supervision section, as well as the sections measuring job satisfaction, excessive work demands, and burnout were transcribed in Microsoft^®^ Excel (Microsoft Corporation, Redmond, WA, USA) and IBM SPSS^®^ (IBM Corporation, Armonk, NY, USA) files. Some values obtained through negatively worded questions in the JSS required transformation to the opposite values. Four binary logistic regressions were conducted to test the significant effect of factors for job satisfaction and burnout (emotional exhaustion, depersonalisation, and personal accomplishment). Across the binary logistic models examining job satisfaction and burnout, the following factors were tested for statistically significant effects: excessive work demands, supervisor support, colleague support, and satisfaction with supervisor.

In line with Plantiveau and colleagues [3], job dissatisfaction was defined as a score between 36 and 108 in JSS [43], while burnout was defined by the following scores in the MBI-ES: above 26 in Emotional Exhaustion, above 5 in Depersonalisation, and below 34 in Personal Accomplishment [50].

### 2.5. Ethical Statement

This study has received ethical approval by the University Ethics Committee (Project identification code SREC126) and has been performed in accordance with the ethical standards as laid down in the 1964 Declaration of Helsinki and its later amendments, and University ethical standards.

## 3. Results

### 3.1. Descriptive Outcomes for Pre-Certification Supervision for BCaBA, BCBA and BCBA-D

A total of 74 respondents provided information on the number of clients served during pre-certification or pre-credentialing supervision, with four respondents reporting having worked with five clients (5.4%), six (8.1%) with four, 26 (35.1%) with three, and 30 (40.5%) with two clients (Table 2). Just below 11% of participants (*n* = 8) reported having worked with only one client during supervision, a practice not permitted under the BACB^®^ experience standards [28].

Participants who did not obtain supervision for free had to pay up to €150 per hour, with the range of prices shown in Table 3.

The majority of the 74 respondents who provided information on the supervisory experience reported being satisfied (87%) with their supervisor. This trend was also reflected when considering the overall evaluation of supervision, with 80% stating they were satisfied (Table 4).

### 3.2. Descriptive and Regression Outcomes for Job Satisfaction, Excessive Work Demands and Burnout

**Figure 1:**
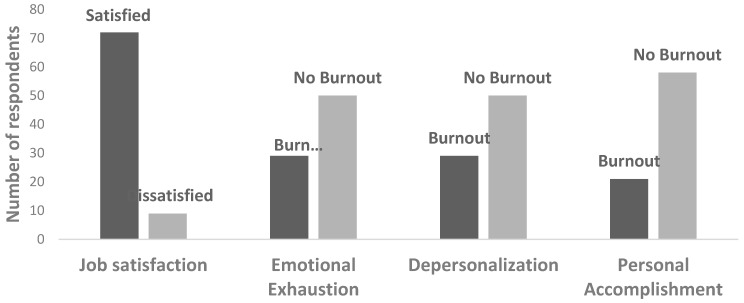
Score distribution in the Maslach burnout inventory domains and the job satisfaction survey (Job Satisfaction: *n* = 81; Burnout: *n* = 79).

A total of 81 respondents completed the JSS section. The majority of respondents were satisfied (89%), whilst the remaining 11% had scores reflecting dissatisfaction (Figure 1). Descriptive statistics highlighted that those aged between 35–44 years had the highest levels of job satisfaction, with 95% obtaining a score reflecting satisfaction. Those aged 45 years or older had the highest proportion of dissatisfaction (15%). When considering colleague support, 87% of those receiving support indicated job satisfaction, whilst 100% of respondents experiencing no colleague support reported having job satisfaction. However, it should be acknowledged that only a small proportion of the 70 respondents providing information on job satisfaction and colleague support were within the no colleague support category (*n* = 7). This small sample size provides an explanation for why job satisfaction was high amongst those receiving no colleague support. This is likely to have affected the results in both the descriptive statistics and regression model. In addition, a total of 70 respondents provided information on both job satisfaction and experience of supervisor support, with analysis indicating that the majority (87%) of respondents who experienced supervisor support had scores reflecting job satisfaction.

In the logistic regression model exploring the effects of excessive work demands, supervisor support, colleague support, and supervisor satisfaction on job satisfaction, only one statistically significant correlation became evident. This model indicated that as the rate of excessive work demands increased, job satisfaction decreased (B = −0.31, S.E. = 0.16, *p* ≤ 0.05). With supervisor support and satisfaction, the opposite trends to those expected were reflected. This model reflected that those receiving no supervisor support or reporting being dissatisfied with their supervisor had higher job satisfaction than those receiving supervisor support or reporting being satisfied with their supervisor; however, these differences were not statistically significant. It is likely that these correlations were in the opposite direction to those expected due to the low cell counts within the no supervisor support/dissatisfied with supervisor categories (*n* = 2 and *n* = 4, respectively). In contrast, those receiving no colleague support had lower scores of job satisfaction compared to those who received colleague support, although this correlation was not statistically significant. Despite colleague support not being statistically significant in this model, its correlation with job satisfaction is in the expected direction, suggesting it may be worth exploring in future research with a larger sample.

In the MBI, 79 respondents provided information on their experience of emotional exhaustion. A total of 63% of respondents experienced no emotional exhaustion burnout in their job, whilst 37% obtained a score reflecting emotional exhaustion burnout (Figure 1). Descriptive analysis indicated that those aged between 35–44 years had the highest levels of emotional exhaustion burnout (68%), whilst those aged 45 years or older had the lowest (31%). In addition, a positive correlation was reflected in the descriptive statistics between colleague support and experiencing no emotional exhaustion burnout (62%). Similar trends were apparent with supervisor support. Those experiencing supervisor support had a higher rate of no emotional exhaustion burnout when compared to those experiencing no supervisor support (61% and 50%, respectively).

The logistic regression model exploring emotional exhaustion burnout indicates only one statistically significant correlation, with those reporting emotional exhaustion burnout being more likely to have higher levels of excessive work demands than those who reported no emotional exhaustion burnout (B = 0.26, S.E. = 0.1, *p* ≤ 0.01). Although all remaining factors in this model were not statistically significant, the correlation with supervisor support indicates that those receiving no supervisor support were more likely to experience emotional exhaustion burnout compared to those receiving supervisor or colleague support. This correlation was also apparent with colleague support; however, it was not statistically significant. Despite their statistical insignificance in this study, these support factors may be of importance to consider in future research with a larger sample.

Moreover, 79 respondents provided information on their experience of depersonalisation in the MBI. As reflected with emotional exhaustion, 63% of respondents experienced no burnout according to depersonalisation, whilst 37% obtained a score reflecting depersonalisation burnout (Figure 1). Descriptive analysis indicated similar levels of depersonalisation burnout across age categories. Of the 68 respondents who provided information on depersonalisation burnout and experience of colleague support, only a small proportion indicated experiencing no colleague or supervisor support (*n* = 7 and *n* = 2, respectively). Overall, a positive correlation was apparent between experiencing colleague support and no depersonalisation burnout (66%). Subsequently, the majority of respondents with no colleague support achieved a score reflecting depersonalisation burnout (71%). Descriptive analysis highlighted that those who experienced no supervisor support had the highest rate of depersonalisation burnout (50%) when compared to those receiving supervisor support (39%).

The logistic regression model indicated that those reporting depersonalisation burnout were more likely to experience higher levels of excessive work demands than those who reported no depersonalisation burnout (B = 0.28, S.E. = 0.11, *p* < 0.01). In addition, no colleague support held a statistically significant correlation with depersonalisation burnout, however in the unexpected direction. The model suggests that those reporting no colleague support were less likely to experience depersonalisation burnout than those with colleague support (B = −2.1, S.E. = 1.04, *p* < 0.05). This may be because only a small number indicated no colleague support, which, therefore, affected the direction and magnitude of the presented correlation. However, as this factor is of statistical significance to depersonalisation burnout, it is worth exploring in future studies with larger sample sizes to verify whether the correlation direction remains the same. In contrast, those who received no supervisor support were more likely to experience depersonalisation burnout than those receiving supervisor support, although this factor was not statistically significant. Again, supervisor support may also be worth considering in future research examining burnout amongst larger sample sizes.

Variation in the MBI was also evident when examining levels of burnout according to personal accomplishment. Of the 79 respondents, 27% gained a score reflecting personal accomplishment burnout, whilst the majority of respondents reflected no burnout (73%) (Figure 1). A negative correlation between age and personal accomplishment burnout was indicated in the descriptive analysis. Those aged between 18 and 34 years had the highest levels of personal accomplishment burnout (32%), when compared to those aged 35–44 (26% experienced burnout) and 45 years or older (8% experienced burnout). As reflected in the depersonalisation descriptive analysis, 68 respondents provided information on their experience of colleague support and personal accomplishment burnout, with a small proportion indicating no experience of colleague support (*n* = 7). Overall, the majority of respondents experiencing colleague support achieved a score of no personal accomplishment burnout (72%). This reflects higher rates of no burnout when compared to other MBI indicators of emotional exhaustion and depersonalisation. In addition, when considering supervisor support, those experiencing no support had the highest levels of personal accomplishment burnout (50%), whilst those receiving support had the lowest rate of personal accomplishment burnout (24%). Similar to those for colleague support, these results reflect higher rates of no burnout when compared to other MBI indicators of emotional exhaustion and depersonalisation.

Although the correlations suggest that those with excessive work demands are more likely to report personal accomplishment burnout, interestingly, the logistic regression model for personal accomplishment indicated these were not statistically significant. Moreover, those receiving no supervisor support or colleague support, respectively, were more likely to experience personal accomplishment burnout than those experiencing supervisor or colleague support. In addition, as expected, those satisfied with their supervisor were less likely to experience burnout according to personal accomplishment when compared to those dissatisfied with their supervisor. Despite none of these correlations being statistically significant, as the direction and magnitude of the presented relationship with personal accomplishment are as expected, it is apparent that these factors may be of importance to explaining personal accomplishment burnout and should therefore be considered in future work with larger sample sizes.

In summary, across all logistic regression models, excessive work demands held a negative correlation with job satisfaction and positive correlations with burnout (Table 5). Consequently, as excessive work demands increased, the rate of burnout according to emotional exhaustion, depersonalisation, and personal accomplishment also increased. These correlations were statistically significant in all models, except for personal accomplishment. Despite this, excessive work demands correlated as expected with all three dimensions of burnout. Moreover, consistency was apparent across analyses in the direction and lack of statistical significance of correlations between burnout and supervisor support. Analyses on job satisfaction reflected that those receiving supervisor support had higher rates of job satisfaction. Analyses exploring the three dimensions of burnout (emotional exhaustion, depersonalisation, and personal accomplishment) indicated that those receiving no supervisor support presented higher levels of burnout than those receiving supervisor support. Slight variation was evident in the direction and statistical significance of correlations when considering burnout rates according to colleague support. In the job satisfaction model, similarly to experience of supervisor support, those receiving colleague support had higher rates of job satisfaction than those receiving no support, however this was not statistically significant. In addition, in the emotional exhaustion and personal accomplishment burnout models, those receiving no colleague support reflected higher rates of burnout, however these correlations were not statistically significant. In contrast, in the depersonalisation model, those receiving no colleague support had significantly lower levels of burnout. It is likely that the low cell count amongst those receiving no colleague support and a depersonalisation burnout score affected this correlation.

## 4. Discussion

This study replicates and expands findings of Plantiveau et al. [3], with the key outcome being that excessive work demands positively correlate with high burnout rates and low job satisfaction. Given the remarkably low numbers of appropriately trained professionals and the frequent lack of funding for behavioural services, both exacerbated outside the USA [51], this outcome, albeit not surprising, would be difficult to overcome. Due to this reality, it might be difficult to reduce actual work demands. However, dedicating a part of supervision to training supervisees’ organisational skills can help remediate the effect of excessive work demands.

First, descriptive results revealed that eight (10.8%) participants worked with only one client during their pre-certification supervision, while another 30 (40.5%) worked with two different clients. In line with the BACB^®^ [28], supervisees must work with at least two different clients during their pre-certification supervised practice. Our finding raises serious concerns, given that working with only one client would not provide supervisees with the necessary skills to serve a highly diverse population once certified. Supervisees should work with at least two, and ideally with more than three different clients, ensuring they acquire a range of skills and generalise these across clients with different characteristics and needs. The BACB^®^ has very recently attempted to streamline the supervision process by making a supervision curriculum mandatory for certificants wishing to supervise students [17], as well as by requesting that supervisors and supervisees complete a 90-min free online training module on supervision since 2015. The latter was then removed in March 2018 [52], therefore an obvious way to remedy lack of compliance with minimum requirements identified in our study might be to re-introduce this training module. Supervisors should also actively seek reassurance that their supervisees comply with this requirement.

Results obtained through the supervision evaluation section strongly suggest that ABA supervision is conducted, overall, in an ethical and professional manner relying on evidence-based practices, therefore resulting in highly satisfied supervisees. Supervision holds a unique position for the dissemination of our science, effective training of younger colleagues, and delivery of quality services to clients. Results of the present study suggest that existing supervisory practices are welcomed by younger professionals, who report being satisfied with their supervisor and the supervisory experience as a whole. Ongoing reliance on evidence-based strategies for supervision will safeguard existing best practice and will allow our field to grow, while keeping ethical standards and professional competence at the forefront.

The rate of burnout lying just above one third of participants for emotional exhaustion and depersonalisation and above one fourth for personal accomplishment is in line with the rates reported in previous studies (e.g., Maslach et al. [1]; Plantiveau et al. [3]), confirming that working in the field of ABA can pose challenges. This outcome is understandable if read in the context of supervisees, who often encounter challenging behaviours, work in intensive one-to-one educational programmes, and lack a support network in countries with few certified professionals. This reality further underscores the need for establishing a support network that bypasses geographical borders, by making use of technology. For example, the use of online blogs that facilitate knowledge transfer, offer support, and empower young professionals through exchange with colleagues from mixed levels of experience could be an ideal solution. Established practices that promote wellbeing among other care professionals could also serve as benchmarks (e.g., Balch et al. [5]).

In turn, job satisfaction appeared to be high, in line with the findings of Plantiveau and colleagues [3] and the expectation that working in the field of ABA brings a feeling that the undertaken tasks are worthwhile and bring good to society. The main aim of professionals working in the field of ABA is to improve the quality of life of the people they serve, an intrinsically high value aim that explains why satisfaction from work has been repeatedly found to be high.

Overall, despite the lack of a statistically significant relationship presented in the logistic regression analysis of this study, the findings suggest that excessive work demands, supervisor support, and colleague support are all important factors influencing job satisfaction and burnout in ABA settings internationally. In line with previous studies (e.g., Gibson et al. [6]; Plantiveau et al. [3]), the descriptive statistics of this study highlight that those receiving support from their supervisor and colleagues were less likely to experience burnout and more likely to report high satisfaction with their job. This outcome is expected, given social support has repeatedly been shown to prevent or alleviate symptoms of burnout [53]. However, the correlations between burnout, job satisfaction, and supervisor and colleague support were not statistically significant in the logistic regression models (i.e., colleague support was only statistically significant in depersonalisation burnout), reflecting the need for a larger sample size to investigate whether the statistical significance of the presented correlations are affected. These findings highlight the importance of building support networks during the course of pre-credentialing supervision that certificants will be able to rely upon after the supervisory experience concludes.

Age, as a potential mediating factor, showed mixed effects. For example, only 12.8% of younger supervisees (18–34-year-olds) reported job dissatisfaction, 59.6% reported emotional exhaustion burnout, 38.3% reported depersonalisation burnout, and 31.9% reported personal accomplishment burnout. In contrast, 4.8% of middle age supervisees (35–44-year-olds) reported job dissatisfaction, 68.4% reported emotional exhaustion burnout, 31.6% reported depersonalisation burnout, and 26.3% reported personal accomplishment burnout. Supervisees with an age of 45 years or higher were the ones most frequently reporting job dissatisfaction (15.4%), and less frequently experiencing emotional exhaustion burnout (30.8%), depersonalisation burnout (38.5%), and personal accomplishment burnout (7.7%), a result that supports the notion that with age comes experience and the skills to protect oneself. In a previous study [3], age was a protective factor against job dissatisfaction and burnout, as older age respondents were less likely to report job dissatisfaction or burnout. Future studies containing larger samples should further explore how age and increasing experience might influence these variables, allowing supervisors to use different strategies according to each group of supervisees’ needs.

## 5. Conclusions

In sum, these findings shed light on the potential of supervision in shaping competent supervisees who not only act in an ethical and professional manner towards clients but also know how to protect themselves from the effect of excessive work demands, risk of burnout, and consequent low level of job satisfaction. Selecting continuous education activities, planning effectively, and actively seeking support networks are all skills that can be learned during the pre-credentialing supervisory experience and require competent and caring supervisors who act as effective teaching agents. Technology can be a unique tool for supervisors to establish cross-country support networks that promote evidence-based practice and offer a rich environment for sharing ethical best practice.

### Implications for Practice

As supervisors seek to train ethical and competent supervisees who will deliver quality services to the community, it is of paramount importance that a range of evidence-based strategies and tools are used. First and foremost, supervisors should explicitly train supervisees on time management and prioritisation of tasks, in order to mitigate the risk of them suffering burnout as a result of excessive work demands.

Overall, supervision training should be based on evidence-based practices, such as the use of operationally defined expectations in terms of client outcomes and supervisee skills, specific feedback, continuous evaluation of staff performance, frequent informal praise, individualised training as per staff member needs, and formal recognition of exceptional performance. Supervisors should be accessible and eager to help with specific staff queries and difficult tasks and strive to maximize the amount of positive interactions they hold with supervisees [54,55].

The purpose of safeguarding supervision contents and minimum standards can be well served by the re-introduction of an interactive training that both supervisors and supervisees must complete before starting the supervision process. Although documentation provides useful details on minimum standards, such as the requirement to work with at least two different clients, technology can help produce more effective tools, such as animated videos, to communicate important information. The requirement to attend continuous professional development training on evidence-based supervisory practice is wisely included in existing regulations and supervisors should ensure that they keep their knowledge up to date with research developments.

Finally, universally accessible support networks that make use of technology should be built, for supervisees to seek advice as needed upon becoming certified. As supervisees are more likely to maintain skills when these are solidly established during the supervision period, supervisors should teach supervisees to make full use of these networks to seek and provide advice to peers, ensuring that direct contact with the benefits of peer-led support groups is experienced early on in their career.

## Figures and Tables

**Table 1:** Outcomes in key demographic variables (*n* = 92).

Variable	Classification	*n* (%)
Gender	Male Female	5 (5%) 87 (95%)
Age	18 to 34 35 to 44 45+	55 (60%) 22 (24%) 15 (16%)
Current work location	North America Europe South America Asia Oceania Africa	46 (50%) 35 (38%) 2 (2%) 5 (5%) 4 (4%) 0 (0%)
Certification status	BCBA-D^®^ BCBA^®^ BCaBA^®^ RBT^®^	5 (5%) 64 (70%) 5 (5%) 18 (20%)

*Notes*. Russia has been classified in Europe, as three out of four verified course sequences in the country are delivered in Moscow, which is located in the European Russian territory. Cyprus has also been classified in Europe.

**Table 2:** Distribution of certificants across number of clients served during pre-credentialing (*n* = 74).

Variable	Classification	*n* (%)
Number of clients served during supervision	1 client 2 clients 3 clients 4 clients 5 clients	8 (10.8%) 30 (40.5%) 26 (35.1%) 6 (8.1%) 4 (5.4%)

**Table 3:** Price per hour of pre-credentialing supervision (*n* = 37).

Variable	Classification	*n* (%)
Price per hour for supervision	0–€49 €50–€74 €75 or more	18 (49%) 12 (32%) 7 (8%)

**Table 4:** Participants’ satisfaction with supervisor and supervision experience.

Overall Evaluation of Supervisor	*n* (%)
Satisfied	64 (87%)
Neither satisfied nor dissatisfied	5 (7%)
Dissatisfied	5 (7%)
Overall evaluation of supervision	
Satisfied	58 (80%)
Neither satisfied nor dissatisfied	9 (12%)
Dissatisfied	6 (8%)

**Table 5:** Binary logistic regression models for Job Satisfaction, Emotional Exhaustion, Depersonalisation, and Personal Accomplishment Burnout.

Model	Independent Variable of Statistical Significance	B
Job Satisfaction	Excessive work demands	−0.31 *
Emotional Exhaustion	Excessive work demands	0.26 **
Depersonalisation	Excessive work demands No colleague support	0.28 ** −2.1*
Personal Accomplishment	none	-

Note: Table 5 reports only the independent variables with statistically significant correlations. *p* < 0.05 * *p* < 0.01 **.
